# Postmortem Appearances of an Old Lady, of 94 Years, Who Had Never Menstruated nor Borne Children

**Published:** 1855-01

**Authors:** A. K. Gardner

**Affiliations:** Member of the National Medical Association, &c.


*Postmortem appearances of an old lady, of* 94 *years, who had never menstruated nor borne children.* By A. K. Gardner, M. D., Member of the National Medical Association, &c.—I was called, about midnight of the 1st of August, 1854, to see Mrs. H——, St. George's Place, attacked with cholera I found her to be an old lady, ninety-four years of age, and in other respects presenting some peculiarities attracting to a medical man. With the ordinary remedies, she recovered perfectly from this attack, but her constitution received a shock from which she never recovered. The day previous she had been walking about her room, and once subsequently she was found, in the middle of the night, sitting in a chair near her bed; with this exception, she never left it subsequently to this attack, except as lifted from it, and placed in a chair for a few minutes. Her appetite failed; and, after a long struggle, of several weeks, with the great conqueror of all, she finally yielded to his supremacy, Oct. 6th; and on the following morning, assisted by my friend, Dr. H. W. Brown, I made a post-mortem at 9 o'clock.

Mrs. H——, although twice married, never had any children. Her second husband was a widower, having a large family by his first wife. More noteworthy was the fact of her having never menstruated, either naturally or vicariously, and her absence of all sexual feelings and appetites.

During the greater part of her life she was troubled with dyspepsia, so that she never indulged in the pleasures of table. Pie-crust and cakes were tabooed articles to her. It was for this delicate condition, supposed to originate from the absence of the menses, that she was advised by her medical men in Massachusetts, where she lived, to marry. This she did; but this natural stimulant to the generative organs produced no local or general amelioration. She subsequently was married to her second husband, whom she also outlived, to act as a mother to a large family.

The entire absence of all sexual propensities, she herself attributed to her strong devotional character, being an attached and ardent member of the Baptist church. In character, Mrs. H. was somewhat acerb, and apt to look upon the moral delinquences of the young with a severe and unpitying eye. In life she was not wanting in activity and promptness, and conducted her household with a prudence and regularity somewhat noted.

The *postmortem examination*, the morning after her decease, disclosed no active or latent disease. The lungs were perfectly healthy, the stomach and other organs in like condition. The generative organs showed an uterus of usual size, and normal in its condition, except that the cavity of one-half was obliterated. The ovaries were both present, although much atrophied; but whether this condition was due to her advanced age or to an original condition, it was impossible to say.

Save a double-fracture of the hip, the last broken and united within a few years, and marked with the peculiar change in the bone, as seen in all the old, nothing else was noticeable.—*N. York Medical Times, December*, 1854.

